# Symptom Structure of Depression and Anxiety in Mothers Following Child Loss: A Network and Bayesian Graph Analysis

**DOI:** 10.1155/da/9965609

**Published:** 2026-04-09

**Authors:** Firoj Al-Mamun, Mohammed A. Mamun, Moneerah Mohammad Almerab, Suzanne Holroyd, David Gozal, Mohammad Muhit

**Affiliations:** ^1^ Department of Public Health, University of South Asia, Dhaka, 1348, Bangladesh; ^2^ CHINTA Research Bangladesh, Savar, Dhaka, 1342, Bangladesh; ^3^ School of Medicine, University of Nottingham, Nottingham, UK, nottingham.ac.uk; ^4^ Department of Psychology, College of Education and Human Development, Princess Nourah Bint Abdulrahman University, Riyadh, Saudi Arabia, pnu.edu.sa; ^5^ Deptartment of Psychiatry and Behavioral Medicine, Joan C. Edwards School of Medicine, Marshall University, 1600 Medical Center Drive, Huntington, 25701, West Virginia, USA, marshall.edu; ^6^ Office of the Dean and Department of Pediatrics, Joan C. Edwards School of Medicine, Marshall University, 1600 Medical Center Drive, Huntington, 25701, West Virginia, USA, marshall.edu

**Keywords:** anxiety, bereavement, depression, directed acyclic graph (DAG), maternal mental health, symptom network analysis

## Abstract

**Background:**

Bereavement, especially following the loss of a child, is a profoundly distressing life event associated with heightened risks of depression and anxiety. However, limited evidence exists on the symptom‐level structure and statistical interrelations of these conditions among bereaved individuals, particularly in low‐ and middle‐income countries (LMICs).

**Methods:**

Using nationally representative data from the 2022 Bangladesh Demographic and Health Survey (BDHS), we identified 2276 bereaved mothers. We applied psychological network analysis to estimate the partial correlation network structure of nine‐item Patient Health Questionnaire (PHQ‐9) depressive and seven‐item Generalized Anxiety Questionnaire (GAD‐7) anxiety symptoms. Centrality, predictability, and bridge metrics were computed. Network comparison tests (NCTs) assessed structural invariance across bereavement subgroups. Bayesian directed acyclic graphs (DAGs) were used to explore conditional dependency patterns and probabilistic edge orientations among symptoms.

**Results:**

The prevalence of probable major depressive disorder (MDD) was 6.69% (95% CI: 5.52–7.86). Prevalence estimates were comparable by bereavement recency, with overlapping confidence intervals among mothers bereaved within the past 3 years (5.34%, 95% CI: 3.06–9.15) and those bereaved more than 3 years earlier (6.83%, 95% CI: 5.69–8.19). The symptom network revealed interconnected domains corresponding to anxiety and depression symptoms. Trouble relaxing and psychomotor disturbance showed the highest strength centrality, while suicidal ideation exhibited the highest predictability in the network. Bridge centrality analysis identified feeling afraid, sadness, irritability, and psychomotor disturbance as the strongest cross‐domain connectors linking anxiety and depression symptoms. Bayesian DAG analysis indicated strong conditional dependencies among worry‐related anxiety symptoms, while psychomotor disturbance showed a strong conditional association with suicidal ideation.

**Conclusions:**

This study offers novel symptom‐level insights into bereavement‐related anxiety and depression among mothers in Bangladesh. The observed symptom patterns are consistent with enduring depressive and anxiety symptoms rather than acute grief alone. Symptom‐focused approaches targeting central and bridge symptoms may support more efficient screening and scalable intervention strategies in bereaved populations.

## 1. Introduction

Bereavement, the experience of losing a loved one, is a profound life stressor that can precipitate intense psychological distress and long‐term mental health consequences [1]. The loss of a close relative, particularly when sudden or unexpected, has been linked to elevated risks of depression, anxiety, posttraumatic stress, and suicidal ideation [2, 3]. While grief is a natural and adaptive response, it can become pathological in some individuals, developing into prolonged grief disorder, major depressive disorder (MDD), or other mood and anxiety‐related conditions [4, 5]. Among bereaved individuals, women, especially mothers, may experience more severe and enduring psychological consequences, often extending years beyond the acute phase of bereavement. This heightened vulnerability may be due to deeper emotional bonds, caregiving roles, and heightened perceptions of responsibility [6, 7].

Among the different types of bereavement, the death of a child is particularly devastating and is often considered one of the most traumatic forms of loss. Parents, especially mothers who experience the death of a child, are at heightened risk of psychiatric morbidity, including MDD, generalized anxiety disorder, and complicated grief [8, 9]. These effects can persist for years, disrupting not only emotional and physical health but also social and familial functioning [6, 10, 11]. Research also indicates that symptom patterns following child loss are influenced by contextual factors such as the age of the deceased child [12] and time since bereavement [9, 10]. Importantly, when clinically significant symptoms persist well beyond the acute phase of bereavement, particularly more than 2 to 3 years postloss, and are accompanied by functional impairment, they may indicate MDD rather than grief‐specific reactions. This distinction has important diagnostic and treatment implications, as persistent depressive and anxiety symptoms may warrant formal psychiatric evaluation, structured screening, and evidence‐based interventions for MDD or anxiety disorders, rather than grief‐focused support alone. However, few studies have systematically examined how these factors shape the symptomatology of MDD and anxiety symptoms at a granular level.

To date, most studies investigating the mental health consequences of bereavement have employed aggregate symptom scores or categorical diagnoses, potentially obscuring important interrelationships between individual symptoms [1, 5, 6, 9, 11, 13]. Network analysis, a data‐driven, psychometric approach that models mental disorders as systems of interacting symptoms, offers a nuanced understanding of psychopathology by identifying central symptoms (those most strongly connected to other symptoms within the network) and bridge symptoms (those that link distinct symptom communities, such as anxiety and depression) [14, 15]. This approach has gained attention in grief and trauma research, where symptom interactions may differ across populations and contexts. While prior network studies have explored the structure of depression or anxiety following bereavement [16–19], few have simultaneously modeled both constructs and assessed directionality between symptoms using causal discovery techniques such as Bayesian network modeling [20]. Directed acyclic graphs (DAGs), for instance, can provide insights into potential dependency structures by identifying symptoms that are probabilistically linked to others.

Despite this growing body of literature, MDD and anxiety remain significantly understudied in low‐ and middle‐income countries (LMICs), including Bangladesh, where maternal bereavement is common but often occurs in contexts of limited mental health support and persistent social stigma, which may be associated with elevated and persistent depressive symptoms [21]. Bangladesh continues to face a dual burden of high child mortality and mental health service underutilization, making maternal bereavement a critical yet overlooked public health issue [22]. Existing studies in Bangladesh have largely focused on perinatal grief or general maternal mental health, with limited attention to how depressive and anxiety symptoms co‐occur and interact in bereaved mothers over time [23]. There is also a lack of research examining whether bereavement characteristics such as recency of loss, age of the deceased child, sex of the child, or number of losses affect symptom patterns and downstream consequences.

To address these gaps, the present study aimed to (i) estimate the prevalence of probable MDD (defined by nine‐item Patient Health Questionnaire [PHQ‐9] ≥ 10) overall and by bereavement recency, (ii) model the symptom‐level network structure of depression and anxiety among bereaved mothers in Bangladesh using data from the nationally representative Bangladesh Demographic and Health Survey (BDHS) 2022, (iii) identify central and bridge symptoms linking the two symptoms, (iv) examine the stability and potential directionality of symptom pathways using Bayesian DAG modeling, and (v) explore how bereavement‐related characteristics may influence the network structure. By integrating psychological network analysis with Bayesian graph modeling, this study provides novel insights into the dynamic architecture of probable MDD and anxiety symptoms in a high‐burden LMIC context. Findings may inform targeted, culturally appropriate interventions to support maternal mental health following child loss in Bangladesh and similar settings.

## 2. Methods

### 2.1. Study Design, Procedure, and Participants

This study utilized data from the BDHS 2022, a nationally representative, cross‐sectional household survey designed to provide estimates of key demographic and health indicators across Bangladesh. Data were collected through structured, interviewer‐administered, face‐to‐face household interviews conducted by trained fieldworkers among women aged 15–49 years. Interviews were conducted in participants’ households using standardized DHS questionnaires, with interviewers recording responses directly. Although responses were not anonymous at the time of data collection, all data were deidentified prior to public release, and strict confidentiality protocols were followed.

The BDHS Women’s Questionnaire was administered in two formats—a long questionnaire (including the mental health module: PHQ‐9 and seven‐item Generalized Anxiety Questionnaire [GAD‐7]) implemented in approximately two‐thirds of sampled households and a short questionnaire in the remaining households. Because the PHQ‐9 and GAD‐7 were included only in the long questionnaire, mental health symptom data were available for a predefined subsample of women.

The BDHS employed a two‐stage stratified cluster sampling design, ensuring coverage of both urban and rural areas across the eight administrative divisions of the country [22]. In the first stage, 675 enumeration areas (EAs) were selected using probability proportional to size—237 from urban and 438 from rural regions. In the second stage, an average of 45 households per EA was systematically sampled. This multistage sampling framework ensured a nationally representative sample of women of reproductive age.

For the present analysis, we focused on bereaved mothers, identified from the BDHS birth history module, which records all live births to interviewed women and the survival status of each child. Child mortality information is captured for up to 20 births per woman, allowing identification of mothers who reported at least one deceased child. Using these birth history records, a total of 3411 mothers were identified as having experienced the loss of at least one child. However, mental health symptom data (i.e., PHQ‐9 and GAD‐7 responses) were available for only 2276 of these women, and thus this subset was retained for the final analytical sample.

All prevalence estimates were weighted using the sampling weights provided by the BDHS to account for the complex survey design, ensuring national representativeness. The analytic sample therefore represents bereaved mothers included in the BDHS mental health module at the population level.

### 2.2. Measures

#### 2.2.1. Bereavement Characteristics

Maternal bereavement was defined using the BDHS birth history module, which records all live births and the survival of each child reported by the mother. For each birth, the mother reports whether the child is alive at the time of interview. A mother was classified as bereaved if she reported at least one child who had died (child survival status recorded as “dead”). In the BDHS dataset, this information is stored across the child survival indicators for up to 20 births (B5$01‐B5$20), where a value of zero indicates the child is deceased. For each deceased child, we retrieved the child’s sex (B4$XX), date of birth (B3$XX), and age at death (B6$XX). Time since death (in months) was derived by subtracting the child’s date of birth and age at death (converted to months) from the mother’s interview date (Century Month Code). Because the BDHS birth history module records only live births and child survival outcomes, the present analysis captures deaths occurring during infancy, childhood, and adolescence but does not include deaths of adult offspring (≥18 years). To ensure one observation per participant, only the most recent child death per mother was retained.

Four bereavement‐related variables were derived to characterize the nature and context of child loss. Recency of loss was categorized as “Recent” (≤36 months) vs. “Long‐term” (>36 months). This cutoff was selected based on bereavement literature indicating that acute grief reactions typically attenuate within the first 2 to 3 years following child loss, whereas the persistence of depressive and anxiety symptoms beyond this period is more consistent with chronic grief reactions or MDD rather than normative bereavement [4, 5, 9, 10]. This cutoff was used for descriptive and comparative purposes and does not imply diagnostic boundaries. Age at death of the deceased child was classified as “Neonatal/Infant” (0–11 months) vs. “Childhood/Older” (≥12 months). Sex of the deceased child was categorized as “Male” vs. “Female.” Number of child losses was classified as “one child” vs. “Two or more children” to distinguish single loss from cumulative bereavement exposure.

#### 2.2.2. Depression and Anxiety Symptoms

Depression was measured using the PHQ‐9 over the past 2 weeks [24]. Each item represents a feature or symptom of depression: anhedonia, sad mood, sleep, energy, appetite, guilt, concentration, motor, and suicide ideation. The responses were recorded on a four‐point Likert scale ranging from zero (never) to three (always). In this study, PHQ‐9 scores ≥ 10 were used to define probable MDD, consistent with established diagnostic screening thresholds. Prevalence estimates were calculated overall and stratified by time since most recent child loss (≤3 years vs. >3 years) to contextualize symptom persistence relative to bereavement duration.

Anxiety was measured using the GAD‐7 over the past 2 weeks [25]. Each item represents a feature or symptom of anxiety: nervousness, uncontrollable worry, excessive worry, trouble relaxing, restlessness, irritability, and fear. The responses were recorded on a four‐point Likert scale ranging from zero (not at all) to three (nearly every day).

The Bangla versions of the PHQ‐9 and GAD‐7 have demonstrated good psychometric validity in prior Bangladeshi studies [26, 27]. A cutoff score of ≥10 has been applied in Bangladeshi studies to identify depressive symptoms [28]. In the present sample of bereaved mothers, both scales showed good internal consistency (Cronbach’s α: PHQ‐9 = 0.82 and GAD‐7 = 0.84).

### 2.3. Ethical Approval

Ethical approval was not required for this study, as it involved secondary analysis of publicly available, deidentified data from the BDHS 2022. The original BDHS survey obtained informed consent from all participants and received ethical clearance from the appropriate institutional review boards [22].

### 2.4. Statistical Analysis

All analyses were conducted in R version 4.4.1. References for R packages used solely for data import or manipulation have been omitted for brevity. Descriptive statistics were used to summarize symptom distributions and bereavement characteristics. Bereaved mothers were first identified from the full sample (*n* = 30,078), yielding 3411 mothers who had experienced the death of at least one child. The PHQ‐9 and GAD‐7 mental health module was administered to a predefined subsample of women (*n* = 19,987). Among bereaved mothers, 2276 belonged to this subsample and therefore had mental health data available. These women comprised the analytic sample.

Sampling weights provided by the BDHS were applied when estimating the weighted prevalence of probable MDD to ensure population‐level representativeness. In contrast, symptom network estimation, network comparison tests (NCTs), and Bayesian DAG analyses were conducted on unweighted data. This approach is consistent with prior psychometric network research, as these methods aim to characterize the relational structure among symptoms rather than produce population prevalence estimates.

#### 2.4.1. Data Preparation

BDHS data were imported, and relevant variables, including bereavement characteristics and 16 symptom items (A1–A7 for anxiety and D1–D9 for depression), were retained. Although PHQ‐9 and GAD‐7 items are scored on a four‐point Likert scale ranging from zero to three, the BDHS dataset also includes nonsubstantive response categories (e.g., refused to answer and do not know), which are coded outside this range. A total of 95 symptom item responses fell outside the valid range and were excluded from analysis. Analyses were restricted to bereaved mothers with complete PHQ‐9 and GAD‐7 item data (MTH1–MTH16) within the mental health module subsample, yielding a final analytic sample of *n* = 2276.

#### 2.4.2. Network Estimation

A regularized partial correlation network was estimated using the Extended Bayesian Information Criterion Graphical Lasso (EBICglasso) algorithm via the bootnet package. This method identifies conditional dependencies between symptoms, controlling for all other symptoms, and applies EBIC model selection to optimize sparsity [29]. The network was visualized using the qgraph package with the Fruchterman–Reingold layout, where nodes represented individual symptoms and edges represented partial correlations. Edge thickness and saturation indicated the strength of relationships. To aid interpretation, nodes were colored by domain: anxiety symptoms (A1–A7) in blue and depression symptoms (D1–D9) in coral.

#### 2.4.3. Centrality, Predictability, and Bridge Centrality

Symptom importance was evaluated using centrality indices (strength, closeness, and betweenness) computed through the centrality() function in qgraph [30]. Strength centrality reflects the overall connectivity of a node with other symptoms and is generally considered the most stable and interpretable centrality metric in psychological networks. Closeness and betweenness centrality were additionally reported to provide complementary information about the relative position of symptoms within the network and their potential role in facilitating connections between symptom clusters. Predictability, reflecting the proportion of variance in each symptom explained by its neighboring nodes, was estimated using the mgm package [31]. To identify cross‐domain connectors, bridge centrality metrics (bridge strength, bridge closeness, and bridge betweenness) were calculated using the networktools package [32], with anxiety and depression symptoms defined as separate communities.

#### 2.4.4. Network Stability and Accuracy

The robustness of the network model was assessed via nonparametric bootstrapping (1000 iterations) using the bootnet package. Edge‐weight accuracy was evaluated by estimating 95% confidence intervals; narrower intervals indicated more reliable edge estimates. Centrality stability was assessed using a case‐dropping bootstrap, with the correlation stability (CS) coefficient quantifying the proportion of the sample that could be dropped while maintaining centrality correlations ≥ 0.7 with the full‐sample estimates. A CS coefficient ≥ 0.5 is considered acceptable, and ≥0.7 is ideal [29].

#### 2.4.5. NCT

Group differences in symptom network structure were assessed using the NetworkComparisonTest package [33]. NCTs were performed across four bereavement‐related subgroups: (i) recency of loss (≤36 months vs. >36 months), (ii) age of the deceased child at death (0–11 months vs. ≥12 months), (iii) sex of the deceased child (male vs. female), and (iv) number of child losses experienced by mother (one child vs. two or more children). Each subgroup comparison was conducted separately for the corresponding bereavement variable. For each subgroup comparison, differences in global strength (overall connectivity) and network structure (invariance) were evaluated using 1000 permutations. Because subgroup comparisons were conducted separately for each bereavement variable, no correction for multiple comparisons was applied. Statistical significance was set at *p*  < 0.05 without correction for multiple comparisons.

### 2.5. Bayesian Network (DAG) Estimation

To examine potential directional relations among symptoms, we conducted Bayesian network analysis using the bnlearn package [34]. All symptom variables were treated as categorical and converted to factors. Structure learning was performed using the hill‐climbing (HC) algorithm with Bayesian Information Criterion (BIC) scoring optimized for discrete data. To ensure robustness, bootstrapping was first performed with 1000 iterations, then increased to 10,000 for the final model [35].

The final consensus DAG was computed by averaging across all bootstrap‐derived networks. Following the procedure recommended by Scutari and Nagarajan [36], we retained only those edges present in at least 50% of bootstrap samples (i.e., threshold = 0.5), a commonly used criterion that balances sensitivity and specificity when identifying stable edges across bootstrap resamples. Edge strength was defined as the proportion of bootstrap samples in which a given edge appeared, and edge directionality was quantified as the probability that a directed edge consistently pointed from one node to another across bootstraps.

The resulting consensus DAG was visualized using the Rgraphviz package. A supplementary visualization highlighted the strongest connections (edge strength > 0.85) visualization [35]. Full edge frequency and direction tables are provided in the Supporting Information.

## 3. Results

### 3.1. Characteristics of the Participants

Among the 2276 bereaved mothers included in the analytic sample, the majority had experienced the death of their most recent child more than 3 years prior to the survey (90.36%, 95% CI: 89.01–91.72). Most mothers reported the loss of a single child (84.21%, 95% CI: 82.64–85.77), while 15.79% (95% CI: 14.23–17.36) had experienced the loss of two or more children.

Regarding characteristics of the deceased child, a slightly higher proportion of losses involved male children (56.51%, 95% CI: 54.37–58.65) compared with female children (43.49%, 95% CI: 41.35–45.63). The majority of deaths occurred during the neonatal or infant period (≤11 months), accounting for 75.24% (95% CI: 73.22–77.27) of first child deaths, whereas 24.76% (95% CI: 22.73–26.78) occurred during later childhood or adolescence. All estimates account for the complex sampling design of the BDHS 2022 (Table 1).

**Table 1:** Descriptive summary of bereavement‐matched sample (*N* = 2276).

Bereavement characteristic	Category	Unweighted *n*	Weighted (%) (95% CI)
Number of child losses	One child	1921	84.21 (82.64–85.77)
	Two or more children	355	15.79 (14.23–17.36)
Sex of deceased child	Male	1271	56.51(54.37–58.65)
	Female	1005	43.49 (41.35–45.63)
Age at first child death	Neonatal/infant (≤11 months)	1717	75.24 (73.22–77.27)
	Childhood/older (≥12 months)	559	24.76 (22.73–26.78)
Recency of most recent child death	≤3 years	229	9.64 (8.28–10.99)
	>3 years	2047	90.36 (89.01–91.72)


*Note:* Percentages are weighted using BDHS 2022 sampling weights and account for the complex survey design.

The mean PHQ‐9 score among bereaved mothers was 3.88 ± 3.58, and the mean GAD‐7 score was 3.74 ± 3.50, indicating generally low to moderate levels of depressive and anxiety symptoms at the population level.

### 3.2. Prevalence of Probable MDD

The weighted prevalence of probable MDD in the study population was 6.69% (95% CI: 5.52%–7.86%). The weighted prevalence of probable MDD was 5.34% (95% CI: 3.06–9.15) among mothers bereaved within the past 3 years and 6.83% (95% CI: 5.69–8.19) among those bereaved more than 3 years earlier. The confidence intervals overlapped substantially, indicating no statistically meaningful difference by recency of bereavement.

### 3.3. Network Structure of Anxiety and Depression Symptoms

**Figure 1:**
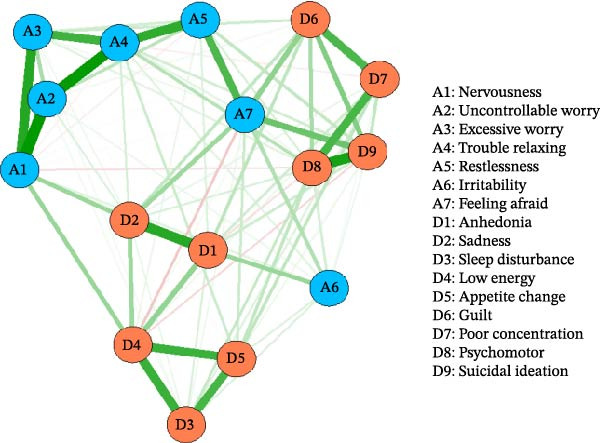
Network structure of anxiety and depression among bereaved mothers.

Figure 1 presents the estimated network structure of anxiety (A1–A7) and depression (D1–D9) symptoms among bereaved mothers. Nodes represent individual symptoms, and edges indicate regularized partial correlations between symptoms after controlling for all others (see Table S1 for correlation matrix). Blue edges represent positive associations, with thickness proportional to the magnitude of the partial correlation.

Within the anxiety domain, the strongest associations were observed between uncontrollable worry (A2) and trouble relaxing (A4) (edge weight = 0.335) and between nervousness (A1) and excessive worry (A3) (0.293). Additional strong within‐domain connections included trouble relaxing (A4) with restlessness (A5) (0.279) and restlessness (A5) and feeling afraid (A7) (0.237). These findings indicate a tightly interconnected cluster of generalized anxiety symptoms, characterized by persistent worry and physiological arousal.

Within the depression domain, strong associations emerged among sleep disturbance (D3), low energy (D4), and appetite change (D5), with edge weights ranging from 0.247 to 0.265. Strong links were also evident between sadness (D2) and anhedonia (D1) (0.290), reflecting a central affective core of depressive symptoms. Additionally, psychomotor disturbance (D8) showed a strong association with suicidal ideation (D9) (0.311), indicating frequent co‐occurrence of these symptoms in the network (see Table S2 for adjacency matrix).

Overall, the network structure demonstrated tightly connected affective, cognitive, and somatic symptoms among bereaved mothers.

### 3.4. Network Centrality and Predictability

Centrality and predictability analyses identified several key symptoms contributing to the structure of anxiety and depression among bereaved mothers (see Figure S1 and Table S3).

Within the anxiety domain, trouble relaxing (A4) emerged as the most central symptom, showing the highest strength (1.258) and expected influence (1.222), indicating its dominant role in the symptom network. Restlessness (A5) and feeling afraid (A7) also demonstrated high centrality, while uncontrollable worry (A2) showed moderate‐to‐high strength, reinforcing the prominence of core worry‐related symptoms.

Within the depression domain, psychomotor disturbance (D8) and low energy (D4) exhibited the highest strength centrality (1.249 and 1.153, respectively), indicating strong connectivity with other depressive symptoms. Sadness (D2) also showed relatively high strength and expected influence, supporting its central role in the depressive symptom network.

Predictability analyses further highlighted suicidal ideation (D9) and psychomotor disturbance (D8) as the most predictable symptoms in the network (0.924 and 0.875, respectively), suggesting that a large proportion of variance in these symptoms is explained by their immediate network neighbors. High predictability was also observed for feeling afraid (A7), poor concentration (D7), and guilt (D6), indicating that these symptoms are tightly embedded within the overall anxiety–depression network.

### 3.5. Network Bridge Centrality

Bridge centrality analysis identified symptoms that most strongly connected the anxiety and depression communities (see Figure S2 and Table S4). Among anxiety symptoms (Community 1), feeling afraid (A7) emerged as the most prominent bridge symptom, exhibiting the highest bridge strength (0.669) and 2‐step bridge expected influence = 0.960. This pattern indicates that fear‐related symptoms occupy a central cross‐domain position, linking anxiety‐related distress with depressive symptoms. Other anxiety symptoms with notable bridging roles included irritability (A6) (bridge strength = 0.356; 2‐step bridge EI = 0.706) and trouble relaxing (A4) (bridge strength = 0.290; 2‐step bridge EI = 0.646), suggesting that physiological arousal and affective dysregulation contribute to cross‐domain symptom connectivity.

Within the depression community (Community 2), sadness (D2) demonstrated the highest bridge strength (0.416) and 2‐step bridge expected influence (0.858), indicating a pivotal role in connecting depressive experiences with anxiety symptoms. Psychomotor disturbance (D8) (bridge strength = 0.314; 2‐step bridge EI = 0.563) and guilt (D6) (bridge strength = 0.263; 2‐step bridge EI = 0.550) also showed elevated bridge metrics, underscoring their relevance in cross‐domain symptom interconnections.

These findings highlight feeling afraid, sadness, irritability, and psychomotor disturbance as salient cross‐domain symptoms that may facilitate co‐occurrence between anxiety and depression among bereaved mothers. Importantly, bridge metrics reflect structural connectivity rather than causal influence and should be interpreted accordingly.

### 3.6. Network Accuracy and Stability

Network stability and accuracy were evaluated using nonparametric bootstrapping (1000 iterations). The CS coefficient for strength centrality was 0.59, exceeding the recommended threshold of 0.50 and indicating acceptable stability of centrality estimates (Figure S3). This suggests that the relative ranking of symptom strength remains reasonably consistent under case‐dropping. Bootstrapped confidence intervals for edge weights (Figure S4) were narrower for stronger edges and wider for weaker ones. This indicates greater precision for the most prominent symptom associations and warranting caution when interpreting connections. Bootstrapped difference tests for strength centrality (Figure S5) showed that highly central symptoms—particularly trouble relaxing (A4), psychomotor disturbance (D8), and suicidal ideation (D9)—were more central than several lower‐strength nodes, though not all pairwise differences were statistically significant. Overall, these findings support the reasonable robustness of the network structure while emphasizing cautious interpretation of small centrality differences.

### 3.7. NCT for Bereavement Variables

NCTs indicated no statistically significant differences in network structure or global strength across any bereavement‐related subgroup comparisons (Table 2). However, these findings should be interpreted cautiously given the substantial imbalance in subgroup sample sizes, particularly for recency of bereavement.

**Table 2:** Network comparison test summary for the bereavement variables.

Comparison group	Test type	Statistic	*p*‐Value	Global strength (group 1)	Global strength (group 2)	Interpretation
Recency of child loss (recent vs. long‐term)	Network structure invariance	*M* = 0.198	0.412	6.649	7.120	No significant difference in network structure or global strength
	Global strength invariance	*S* = 0.470	0.113	—	—	
Age at child death (neonatal/infant vs. childhood/older)	Network structure invariance	*M* = 0.127	0.748	7.095	6.884	No significant difference in network structure or global strength
	Global strength invariance	*S* = 0.212	0.213	—	—	
Sex of the deceased child (male vs. female)	Network structure invariance	*M* = 0.121	0.617	7.053	6.998	No significant difference in network structure or global strength
	Global strength invariance	*S* = 0.055	0.688	—	—	
Number of child losses (one vs. multiple children)	Network structure invariance	*M* = 0.179	0.308	7.209	6.938	No significant difference in network structure or global strength
	Global strength invariance	*S* = 0.271	0.202	—	—	

### 3.8. DAG Analysis

**Figure 2:**
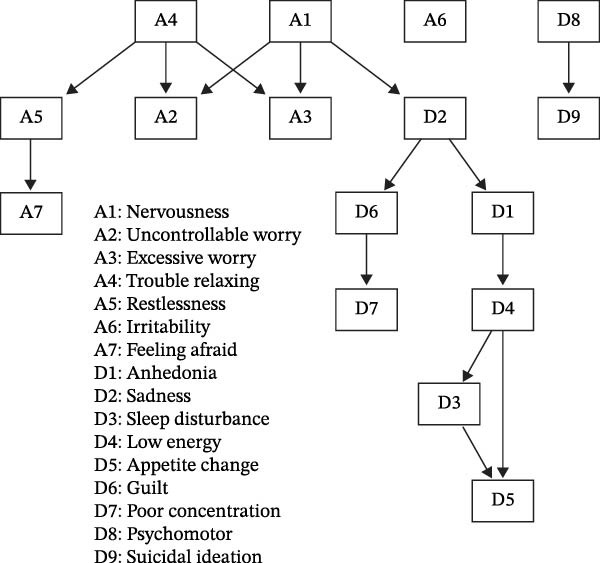
A directed acyclic graph (DAG) of anxiety and depression symptoms among bereaved mothers.

The Bayesian network, estimated via a bootstrapped HC algorithm, was used to explore conditional dependencies among anxiety and depression symptoms (Figure 2). Edges represent conditional dependencies, with edge strength indicating the proportion of bootstrap samples in which the edge was present, and direction reflecting the relative frequency of edge orientation across resamples.

Within the anxiety symptom cluster, trouble relaxing (A4) showed strong conditional associations with uncontrollable worry (A2), excessive worry (A3), and restlessness (A5), with edge strengths exceeding 0.96. Nervousness (A1) showed strong conditional associations with both uncontrollable worry and excessive worry, indicating a tightly interrelated worry‐arousal symptom constellation. Restlessness was also strongly associated with feeling afraid (A7).

Within depression cluster, sadness (D2) was associated with anhedonia (D1). Low energy (D4) was connected with sleep disturbance (D3) and appetite change (D5), reflecting a coherent somatic symptom pattern. Psychomotor disturbance (D8) and suicidal ideation (D9) also showed a strong association. Although the edge was slightly more frequently oriented from D8 to D9 (direction probability = 0.55), this does not provide sufficient evidence to infer a reliable directional or upstream relationship.

**Figure 3:**
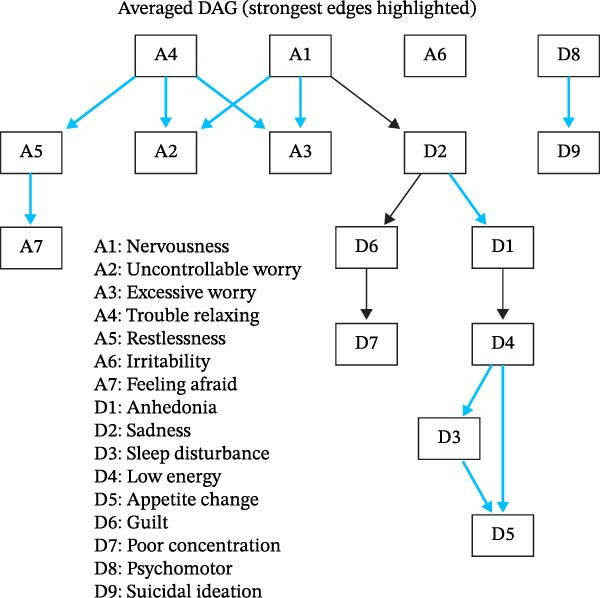
Directed acyclic graph (DAG). Edges signify the importance (BIC value) of the edge to model fit.

Edge strengths across the DAG were predominantly high (≥0.85), indicating stable symptom dependencies across bootstrap samples (Figure 3; Table S5). However, given the cross‐sectional nature of the data and the limited certainty of edge orientation for several symptom pairs, the DAG results should be interpreted as exploratory and hypothesis‐generating rather than causal.

## 4. Discussion

This study is one of the first to explore the symptom‐level structure and probabilistic associations of anxiety and depression among bereaved mothers in a nationally representative sample, utilizing both undirected network analysis and Bayesian DAG modeling. Results suggested that the prevalence of probable MDD in this population was 6.69%. Prevalence estimates by recency showed overlapping confidence intervals (≤3 years: 5.34% [95% CI: 3.06–9.15] vs. >3 years: 6.83% [95% CI: 5.69–8.19]), indicating no statistically meaningful difference by time since loss. This lack of differentiation by bereavement recency may reflect the persistent and enduring nature of grief‐related psychopathology, which does not necessarily resolve within a fixed timeframe. Given that most mothers were more than 3 years postloss (90.36%), clinically relevant symptoms observed in this sample are likely to reflect enduring depressive and anxiety symptomatology rather than acute grief alone. In the Bangladeshi context, limited access to mental health services and ongoing social stressors may further contribute to symptom persistence among bereaved mothers. This interpretation is consistent with evidence that a subset of bereaved individuals experiences persistent distress beyond the early bereavement period [6].

The psychological network structure observed in this study reflects the multifaceted and interconnected nature of bereavement‐related psychopathology. Within the anxiety domain, the strongest associations were identified between uncontrollable worry and trouble relaxing and between nervousness and excessive worry. Additional strong within‐anxiety connections (trouble relaxing‐restlessness and restlessness‐feeling afraid) further suggest that worry‐related symptoms tend to co‐occur closely with physiological arousal and fear‐related experiences. This configuration aligns with prior network studies highlighting the central role of worry and hyperarousal in anxiety disorders [37]. Similarly, the depression network revealed a prominent somatic triad comprising sleep disturbances, low energy, and appetite changes, echoing prior findings where vegetative symptoms cluster closely in depressive states [38, 39]. The strong association between sadness and anhedonia highlights an affective core that may characterize depressive experiences among bereaved mothers. Importantly, the strong association between psychomotor disturbance and suicidal ideation indicates these symptoms frequently co‐occur, which may be important for suicide risk monitoring [39]. Together, these findings reinforce the utility of symptom network models in capturing nuanced interrelations often overlooked by traditional latent variable approaches, offering a more dynamic understanding of mental health responses following child loss.

Centrality and predictability analyses provide insight into the hierarchical organization of symptoms and the relative importance of individual nodes within the network. Among anxiety symptoms, trouble relaxing, restlessness, and uncontrollable worry emerged as the most central, consistent with previous network studies emphasizing arousal and worry‐related symptoms in anxiety disorders [37, 39, 40]. Within the depression cluster, psychomotor disturbance, low energy, and sadness showed the highest centrality, consistent with previous studies highlighting the somatic and affective symptoms in depressive networks [37, 39, 40]. Predictability was highest for suicidal ideation and psychomotor disturbance, indicating that these symptoms are strongly explained by their immediate network neighbors. This suggests that these symptoms are structurally embedded within the broader anxiety‐depression system and reflect downstream endpoint symptom. However, high predictability should not be interpreted as causal dependence but rather as statistical interconnectedness within the network.

Bridge centrality analysis identified feeling afraid and sadness as key connectors between anxiety and depression domains. The prominence of feeling afraid as a bridge symptom, consistent with previous studies [39], may reflect the co‐occurrence of fear‐related distress with depressive symptoms following child loss [41]. Within the depression community, sadness as a bridge symptom highlights its capacity to link emotional, cognitive, and somatic dimensions of mental distress [39, 42]. The identification of psychomotor disturbance as additional bridging symptoms suggests that motoric slowing may facilitate the co‐occurrence of anxiety and depression symptomatology [40]. Clinically, these bridging nodes may represent efficient intervention targets, as addressing them may help attenuate cross‐domain symptom clustering [32, 43]. However, bridge indices reflect patterns of structural connectivity rather than causal influence and should be interpreted accordingly.

The NCTs did not detect statistically significant differences in either global strength or overall network structure across bereavement‐related subgroups. Although these findings suggest broadly similar symptom interrelations across groups, they should be interpreted with caution. In particular, the comparison by recency of bereavement involved markedly unequal sample sizes, with a substantially smaller recently bereaved group, limiting statistical power and increasing the risk of Type II error. Consequently, the absence of statistically significant differences should not be interpreted as definitive evidence of structural invariance. Rather, these findings indicate that no detectable differences were observed under the current sample conditions. Nevertheless, the largely comparable network patterns observed across subgroups align with prior network studies reporting relative stability of depression and anxiety symptom structures across subpopulations, such as gender [39, 44]. From a clinical perspective, these results tentatively suggest that highly central and bridge symptoms, such as feeling afraid and sadness, may represent broadly relevant intervention targets across different bereavement contexts, although confirmation in future studies with more balanced subgroup samples is warranted.

The Bayesian DAG analysis complemented the undirected network by estimating conditional dependency patterns among symptoms. Within the anxiety cluster, trouble relaxing showed strong conditional associations with uncontrollable worry, excessive worry, and restlessness. This pattern supports theoretical models of generalized anxiety wherein physiological arousal and tension co‐occur with worry‐related symptoms [41]. Similarly, nervousness was conditionally associated with core worry symptoms, reinforcing its role in broader anxiety manifestations. These findings align with prior DAG‐based models that emphasize arousal‐related symptoms as early or central components within anxiety symptom network [35, 45]. Within the depression domain, sadness was conditionally associated with anhedonia, and low energy was connected with sleep disturbance and appetite changes, reflecting coherent somatic‐affective patterning. Psychomotor disturbance and suicidal ideation showed a strong conditional association. However, the modest direction probability does not support a reliable directional or “upstream” interpretation. More broadly, although several edges showed high strength across bootstrap samples, uncertainty in edge orientation combined with cross‐sectional data suggests the DAG findings should be interpreted as exploratory and hypothesis‐generating rather than causal.

## 5. Practical Implications

This study advances the existing bereavement literature by moving beyond prevalence estimates and aggregate symptom scores to identify symptom‐level mechanisms that structure comorbid anxiety and depression among bereaved mothers in Bangladesh. By applying network and Bayesian graph approaches, the findings highlight a small set of highly central and bridge symptoms, particularly trouble relaxing, sadness, feeling afraid, and psychomotor disturbance, that appear to organize broader patterns of psychological distress following child loss. From a clinical and public health perspective, these results suggest that symptom‐focused screening strategies may be more efficient than disorder‐level assessments alone, especially in low‐resource settings. Brief screening tools that prioritize these influential symptoms may improve early identification of mothers at risk for persistent or comorbid mental health problems, even when full diagnostic assessment is not feasible.

The largely similar network structures observed across bereavement subgroups tentatively suggest that these symptom targets are broadly relevant regardless of recency of loss, age at death, or number of losses, supporting the universal, scalable intervention approaches rather than highly stratified programs. This is particularly important in Bangladesh, where access to specialist mental health services is limited and maternal mental health care is often integrated into general health or community‐based services.

Although the DAG analysis does not permit causal inference, the observed conditional dependencies point to symptoms related to physiological arousal and affective distress as structurally embedded within wider symptom constellations. These symptoms may represent practical priorities for early psychosocial intervention, with the goal of preventing escalation into more severe or comorbid symptom profiles.

These findings have direct relevance for task‐sharing and nonspecialist mental health care models. Community health workers, midwives, and primary‐care providers could be trained to recognize and respond to central and bridge symptoms using brief, evidence‐based psychosocial strategies within existing maternal and child health platforms. Incorporating symptom‐network–informed perspectives into bereavement care guidelines may enhance the precision, efficiency, and reach of mental health services for grieving mothers in Bangladesh and similar low‐resource contexts.

## 6. Strength, Limitations, and Future Directions

This study offers several notable strengths. First, it is one of the first to apply both undirected network analysis and Bayesian DAG modeling to examine symptom‐level associations between anxiety and depression among bereaved mothers using nationally representative data. Second, the large overall sample size enhances generalizability, while the use of complementary analytic techniques provides a nuanced understanding of both symptom interconnections and potential conditional dependencies. Third, although subgroup analyses should be interpreted cautiously, the broadly consistent pattern of central and bridge symptoms across bereavement‐related characteristics suggests potential robustness of key symptom targets across diverse maternal experiences of child loss.

However, several limitations should be acknowledged. The cross‐sectional nature of the data limits causal inference and temporal validation of the directed edges inferred from the DAG models. Additionally, the reliance on interviewer‐administered, self‐reported symptom data may be subject to recall or social desirability bias, particularly in sociocultural contexts where mental health stigma remains pervasive. Importantly, subgroup analyses, especially those stratified by recency of bereavement, were affected by substantial sample imbalance, which may have reduced power to detect true differences in network structure. As a result, null findings from NCTs should be viewed as inconclusive rather than confirmatory.

Although the study population is described as “bereaved mothers,” the majority of participants were more than 3 years postloss. According to DSM‐5 criteria, the persistence of symptoms such as sleep disturbance, fatigue, appetite change, psychomotor changes, suicidal ideation, and anxiety beyond the acute bereavement period is more consistent with probable MDD than uncomplicated grief. Thus, the present findings likely reflect symptom networks of probable MDD with co‐occurring anxiety among bereaved mothers, rather than grief‐specific symptomatology.

Future research should prioritize longitudinal and adequately powered subgroup designs to clarify temporal dynamics and test whether network structures differ meaningfully across stages of bereavement. Integrating biological or contextual variables, such as cortisol levels, social support, or bereavement rituals, may further elucidate mechanisms underlying symptom persistence. Finally, intervention studies targeting the central and bridge symptoms identified here could help evaluate the clinical utility of network‐informed approaches for prevention and treatment among bereaved mothers.

## 7. Conclusion

This study provides nationally representative, symptom‐level evidence on co‐occurring anxiety and depression among bereaved mothers in Bangladesh using network Bayesian graph approaches. The prevalence of probable MDD was 6.69%, and prevalence by bereavement recency showed overlapping confidence intervals, indicating no statistically meaningful difference between mothers bereaved ≤3 years versus >3 years. Symptom network analyses identified trouble relaxing, restlessness, feeling afraid, sadness, psychomotor disturbance, and low energy as highly central and/or cross‐domain bridge symptoms, while psychomotor disturbance and suicidal ideation showed strong co‐occurrence and high predictability. DAG findings suggested stable conditional dependencies but did not support reliable directional (“upstream”) inferences, reinforcing that results are exploratory and hypothesis‐generating.

These findings support symptom‐focused screening and scalable, task‐shared psychosocial interventions that prioritize fear‐related distress, physiological arousal/tension, and core affective‐somatic depressive symptoms within existing maternal and primary‐care platforms in Bangladesh while emphasizing the need for longitudinal studies to clarify symptom dynamics across the bereavement trajectory.

## Funding

Moneerah Mohammad Almerab is currently receiving funding support from the Princess Nourah Bint Abdulrahman University Researchers supporting Project Number PNURSP2026R563, Princess Nourah Bint Abdulrahman University, Riyadh, Saudi Arabia.

## Conflicts of Interest

The authors declare no conflicts of interest.

## Supporting Information

Additional supporting information can be found online in the Supporting Information section.

## Supporting information


**Supporting Information** Table S1: Polychoric correlation matrix for anxiety and depression symptoms among bereaved mothers (*n* = 2276). Table S2: Adjacency (edge‐weight) matrix from EBICglasso network of anxiety and depression symptoms among bereaved mothers (*n* = 2276). Table S3: Centrality and predictability of anxiety and depression symptoms among bereaved mothers (*n* = 2276). Table S4: Bridge centrality metrics for anxiety and depression symptoms among bereaved mothers (*n* = 2276). Table S5: Strongest directed edges in the Bayesian network (DAG) estimated from bootstrapped hill‐climbing algorithm (bnlearn), ordered by edge frequency (strength > 0.85). For each edge, strength represents the proportion of bootstrap samples in which the edge appeared, and direction indicates the proportion of times the edge was oriented in the depicted direction (from → to). Figure S1: Centrality measures for anxiety and depression among bereaved mothers. Figure S2: Bridge centrality measures for anxiety and depression among bereaved mothers. Figure S3: Network stability (e.g., strength) for anxiety and depression symptoms by case‐dropping subset bootstrap. Figure S4: Bootstrap confidence intervals for edge weights. Each line represents the variability (95% CI) of an edge weight across 1000 bootstrap samples, with stronger edges showing narrower intervals and higher stability. Figure S5: Bootstrapped difference tests (*α* = 0.05) results between the node strength of depression and anxiety symptoms. (Note: The black boxes indicate a significant difference between the nodes, and the gray boxes indicate no significant difference between the nodes. White boxes show the values of node strength).

## Data Availability

The deidentified BDHS datasets are publicly accessible upon request from the DHS Program website (https://dhsprogram.com).

## References

[1] Herbert D. , Young K. , Pietrusińska M. , and MacBeth A. , The Mental Health Impact of Perinatal Loss: A Systematic Review and Meta-Analysis, Journal of Affective Disorders. (2022) 297, 118–129, 10.1016/j.jad.2021.10.026. 34678403

[2] Lannen P. K. , Wolfe J. , Prigerson H. G. , Onelov E. , and Kreicbergs U. C. , Unresolved Grief in a National Sample of Bereaved Parents: Impaired Mental and Physical Health 4 to 9 Years Later, Journal of Clinical Oncology. (2008) 26, no. 36, 5870–5876, 10.1200/JCO.2007.14.6738, 2-s2.0-58049203002. 19029425 PMC2645112

[3] Stroebe M. , Schut H. , and Stroebe W. , Health Outcomes of Bereavement, The Lancet. (2007) 370, no. 9603, 1960–1973, 10.1016/S0140-6736(07)61816-9, 2-s2.0-36549007003. 18068517

[4] Shear M. K. , Simon N. , and Wall M. , et al.Complicated Grief and Related Bereavement Issues for DSM-5, Depression and Anxiety. (2011) 28, no. 2, 103–117, 10.1002/da.20780, 2-s2.0-79551625648. 21284063 PMC3075805

[5] Kristensen P. , Weisaeth L. , and Heir T. , Bereavement and Mental Health After Sudden and Violent Losses: A Review, Psychiatry: Interpersonal and Biological Processes. (2012) 75, no. 1, 76–97, 10.1521/psyc.2012.75.1.76, 2-s2.0-84861014418. 22397543

[6] Pohlkamp L. , Kreicbergs U. , and Sveen J. , Bereaved Mothers’ and Fathers’ Prolonged Grief and Psychological Health 1 to 5 Years After Loss—A Nationwide Study, Psycho-Oncology. (2019) 28, no. 7, 1530–1536, 10.1002/pon.5112, 2-s2.0-85067622939. 31108000

[7] Youngblut J. M. and Brooten D. , Comparison of Mothers and Grandmothers Physical and Mental Health and Functioning Within 6 Months After Child NICU/PICU Death, Italian Journal of Pediatrics. (2018) 44, no. 1, 10.1186/s13052-018-0531-8, 2-s2.0-85051318862. PMC6086060 30097046

[8] Rogers C. H. , Floyd F. J. , Seltzer M. M. , Greenberg J. , and Hong J. , Long-Term Effects of the Death of a Child on Parents, Journal of Family Psychology. (2008) 22, no. 2, 203–211, 10.1037/0893-3200.22.2.203, 2-s2.0-43849100903. 18410207 PMC2841012

[9] Redican E. , Shevlin M. , Hyland P. , Murphy J. , Duffy M. , and Karatzias T. , The Psychological Burden of Bereavement in the General Population of UK and Ireland, Death Studies. (2026) 50, no. 2, 277–285, 10.1080/07481187.2024.2420877. 39470747

[10] Youngblut J. A. M. , Brooten D. , Cantwell G. P. , Del Moral T. , and Totapally B. , Parent Health and Functioning 13 Months After Infant or Child NICU/PICU Death, Pediatrics. (2013) 132, no. 5, e1295–e1301, 10.1542/peds.2013-1194, 2-s2.0-84887037909. 24101760 PMC3813397

[11] Zhao X. , Hu H. , Zhou Y. , and Bai Y. , What Are the Long-Term Effects of Child Loss on Parental Health? Social Integration as Mediator, Comprehensive Psychiatry. (2020) 100, 10.1016/j.comppsych.2020.152182, 152182. 32430143

[12] Zetumer S. , Young I. , and Shear M. K. , et al.The Impact of Losing a Child on the Clinical Presentation of Complicated Grief, Journal of Affective Disorders. (2015) 170, 15–21, 10.1016/j.jad.2014.08.021, 2-s2.0-84907943576. 25217759 PMC4253869

[13] Baumann I. , Künzel J. , Goldbeck L. , Tutus D. , and Niemitz M. , Posttraumatic Stress, and Depression Among Bereaved Parents: Prevalence and Response to an Intervention Program, OMEGA - Journal of Death and Dying. (2022) 84, no. 3, 837–855, 10.1177/0030222820918674. 32290762

[14] Borsboom D. , A Network Theory of Mental Disorders, World Psychiatry. (2017) 16, no. 1, 5–13, 10.1002/wps.20375, 2-s2.0-85010674507. 28127906 PMC5269502

[15] McNally R. J. , Can Network Analysis Transform Psychopathology?, Behaviour Research and Therapy. (2016) 86, 95–104, 10.1016/j.brat.2016.06.006, 2-s2.0-84978841949. 27424882

[16] Fried E. I. , van Borkulo C. D. , Cramer A. O. J. , Boschloo L. , Schoevers R. A. , and Borsboom D. , Mental Disorders as Networks of Problems: A Review of Recent Insights, Social Psychiatry and Psychiatric Epidemiology. (2017) 52, no. 1, 1–10, 10.1007/s00127-016-1319-z, 2-s2.0-85001976709. 27921134 PMC5226976

[17] Xiong J. , Chen Z. , and Ma H. , et al.Network Analysis of Prolonged Grief Disorder and Anxiety Symptoms Among Bereaved Chinese Parents Who Lost Their Only Child, Death Studies. (2026) 50, no. 3, 436–447, 10.1080/07481187.2024.2432301. 39589731

[18] Jiang W. , Qian W. , Xie T. , Yu X. , Liu X. , and Wang J. , Patterns and Relationships of Prolonged Grief, Post-Traumatic Stress, and Depressive Symptoms in Chinese Shidu Parents: Latent Profile and Network Analyses, Death Studies. (2026) 50, no. 2, 248–262, 10.1080/07481187.2024.2420242. 39495625

[19] Ma H. , Zhao S. , and Wang Y. , The Symptom Structure of Depression, Anxiety and Suicidal Ideation Among Chinese Shidu Parents—A Network Analysis, Clinical Psychology & Psychotherapy. (2025) 32, no. 1, 10.1002/cpp.70042. 39887507

[20] Malgaroli M. , Maccallum F. , and Bonanno G. A. , Symptoms of Persistent Complex Bereavement Disorder, Depression, and PTSD in a Conjugally Bereaved Sample: A Network Analysis, Psychological Medicine. (2018) 48, no. 14, 2439–2448, 10.1017/S0033291718001769, 2-s2.0-85049955638. 30017007

[21] Shaohua L. and Shorey S. , Psychosocial Interventions on Psychological Outcomes of Parents With Perinatal Loss: A Systematic Review and Meta-Analysis, International Journal of Nursing Studies. (2021) 117, 10.1016/j.ijnurstu.2021.103871, 103871. 33548593

[22] National Institute of Population Research and Training (NIPORT) and ICF , Bangladesh Demographic and Health Survey 2022: Key Indicators Report, 2023, https://dhsprogram.com/pubs/pdf/PR148/PR148.pdf.

[23] Gausia K. , Moran A. C. , Ali M. , Ryder D. , Fisher C. , and Koblinsky M. , Psychological and Social Consequences Among Mothers Suffering From Perinatal Loss: Perspective From a Low Income Country, BMC Public Health. (2011) 11, no. 1, 1–9, 10.1186/1471-2458-11-451, 2-s2.0-79958065881. 21658218 PMC3124431

[24] Kroenke K. , Spitzer R. L. , and Williams J. B. W. , The PHQ-9: Validity of a Brief Depression Severity Measure, Journal of General Internal Medicine. (2001) 16, no. 9, 606–613, 10.1046/j.1525-1497.2001.016009606.x, 2-s2.0-0034853189. 11556941 PMC1495268

[25] Spitzer R. L. , Kroenke K. , Williams J. B. W. , and Löwe B. , A Brief Measure for Assessing Generalized Anxiety Disorder: The GAD-7, Archives of Internal Medicine. (2006) 166, no. 10, 1092–1097, 10.1001/archinte.166.10.1092, 2-s2.0-33646815612. 16717171

[26] Naher R. , Rabby M. R. A. , and Sharif F. , Validation of Patient Health Questionnaire-9 for Assessing Depression of Adults in Bangladesh, Dhaka University Journal of Biological Sciences. (2021) 30, no. 2, 275–281, 10.3329/dujbs.v30i2.54652.

[27] Dhira T. A. , Rahman M. A. , Sarker A. R. , Mehareen J. , and Innamorati M. , Validity and Reliability of the Generalized Anxiety Disorder-7 (GAD-7) Among University Students of Bangladesh, PLoS ONE. (2021) 16, no. 12, 10.1371/journal.pone.0261590. PMC8675645 34914811

[28] Rasheduzzaman M. , al Mamun F. , Faruk M. O. , Hosen I. , and Mamun M. A. , Depression in Bangladeshi University Students: The Role of Sociodemographic, Personal, and Familial Psychopathological Factors, Perspectives in Psychiatric Care. (2021) 57, no. 4, 1585–1594, 10.1111/ppc.12722. 33442872

[29] Epskamp S. , Borsboom D. , and Fried E. I. , Estimating Psychological Networks and Their Accuracy: A Tutorial Paper, Behavior Research Methods. (2018) 50, no. 1, 195–212, 10.3758/s13428-017-0862-1, 2-s2.0-85016010727. 28342071 PMC5809547

[30] Epskamp S. , Cramer A. O. J. , Waldorp L. J. , Schmittmann V. D. , and qgraph Borsboom D. , Network Visualizations of Relationships in Psychometric Data, Journal of Statistical Software. (2012) 48, no. 4, 1–18, 10.18637/jss.v048.i04.

[31] Haslbeck J. M. B. and Waldorp L. J. , mgm: Estimating Time-Varying Mixed Graphical Models in High-Dimensional Data, Journal of Statistical Software. (2020) 93, no. 8, 1–46, 10.18637/jss.v093.i08.

[32] Jones P. J. , Ma R. , and McNally R. J. , Bridge Centrality: A Network Approach to Understanding Comorbidity, Multivariate Behavioral Research. (2021) 56, no. 2, 353–367, 10.1080/00273171.2019.1614898, 2-s2.0-85067660019. 31179765

[33] van Borkulo C. D. , van Bork R. , and Boschloo L. , et al.Comparing Network Structures on Three Aspects: A Permutation Test, Psychological Methods. (2023) 28, no. 6, 1273–1285, 10.1037/met0000476. 35404628

[34] Scutari M. , Learning Bayesian Networks With the Bnlearn R Package, Journal of Statistical Software. (2010) 35, no. 3, 1–22, 10.18637/jss.v035.i03, 2-s2.0-77955124773.21603108

[35] McNally R. J. , Heeren A. , and Robinaugh D. J. , A Bayesian Network Analysis of Posttraumatic Stress Disorder Symptoms in Adults Reporting Childhood Sexual Abuse, European Journal of Psychotraumatology. (2017) 8, no. sup3, 10.1080/20008198.2017.1341276. PMC5632780 29038690

[36] Scutari M. and Nagarajan R. , Identifying Significant Edges in Graphical Models of Molecular Networks, Artificial Intelligence in Medicine. (2013) 57, no. 3, 207–217, 10.1016/j.artmed.2012.12.006, 2-s2.0-84876705387. 23395009 PMC4070079

[37] Beard C. , Millner A. J. , and Forgeard M. J. C. , et al.Network Analysis of Depression and Anxiety Symptom Relationships in a Psychiatric Sample, Psychological Medicine. (2016) 46, no. 16, 3359–3369, 10.1017/S0033291716002300, 2-s2.0-84987673606. 27623748 PMC5430082

[38] Fried E. I. , Epskamp S. , Nesse R. M. , Tuerlinckx F. , and Borsboom D. , What Are “Good” Depression Symptoms? Comparing the Centrality of DSM and Non-DSM Symptoms of Depression in a Network Analysis, Journal of Affective Disorders. (2016) 189, 314–320, 10.1016/j.jad.2015.09.005, 2-s2.0-84943609022. 26458184

[39] Luo J. , Bei D. L. , and Zheng C. , et al.The Comorbid Network Characteristics of Anxiety and Depressive Symptoms Among Chinese College Freshmen, BMC Psychiatry. (2024) 24, no. 1, 1–11, 10.1186/s12888-024-05733-z. 38641813 PMC11027377

[40] Xu S. , Ju Y. , and Wei X. , et al.Network Analysis of Suicide Ideation and Depression–Anxiety Symptoms Among Chinese Adolescents, General Psychiatry. (2024) 37, no. 2, 10.1136/gpsych-2023-101225. PMC10982688 38562407

[41] Craske M. G. , Rauch S. L. , Ursano R. , Prenoveau J. , Pine D. S. , and Zinbarg R. E. , What Is an Anxiety Disorder?, Depression and Anxiety. (2009) 26, no. 12, 1066–1085, 10.1002/da.20633, 2-s2.0-73249125597. 19957279

[42] Zhang W. , Liu T. , Leung D. K. Y. , Chan S. , Wong G. , and Lum T. , Sad Mood Bridges Depressive Symptoms and Cognitive Performance in Community-Dwelling Older Adults: A Network Approach, Innovation in Aging. (2024) 8, no. 1, 10.1093/geroni/igad139, igad139. 38351984 PMC10863485

[43] Robinaugh D. J. , Hoekstra R. H. A. , Toner E. R. , and Borsboom D. , The Network Approach to Psychopathology: A Review of the Literature 2008–2018 and an Agenda for Future Research, Psychological Medicine. (2020) 50, no. 3, 353–366, 10.1017/S0033291719003404. 31875792 PMC7334828

[44] Cai H. , Bai W. , and Liu H. , et al.Network Analysis of Depressive and Anxiety Symptoms in Adolescents During the Later Stage of the COVID-19 Pandemic, Translational Psychiatry. (2022) 12, no. 1, 1–8, 10.1038/s41398-022-01838-9. 35273161 PMC8907388

[45] McNally R. J. , Robinaugh D. J. , Wu G. W. Y. , Wang L. , Deserno M. K. , and Borsboom D. , Mental Disorders as Causal Systems, Clinical Psychological Science. (2015) 3, no. 6, 836–849, 10.1177/2167702614553230, 2-s2.0-84958165455.

