# Supplementary material for: Symptom Structure of Depression and Anxiety in Mothers Following Child Loss: A Network and Bayesian Graph Analysis
Source: Depress Anxiety. 2026 Apr 9;2026:9965609. doi: 10.1155/da/9965609 (PMC13062755; doi:10.1155/da/9965609)
Supplement: Supplementary file 1 — Supporting Information Table S1: Polychoric correlation matrix for anxiety and depression symptoms among bereaved mothers (n = 2276). Table S2: Adjacency (edge‐weight) matrix from EBICglasso network of anxiety and depression symptoms among bereaved mothers (n = 2276). Table S3: Centrality and predictability of anxiety and depression symptoms among bereaved mothers (n = 2276). Table S4: Bridge centrality metrics for anxiety and depression symptoms among bereaved mothers (n = 2276). Table S5: Strongest directed edges in the Bayesian network (DAG) estimated from bootstrapped hill‐climbing algorithm (bnlearn), ordered by edge frequency (strength > 0.85). For each edge, strength represents the proportion of bootstrap samples in which the edge appeared, and direction indicates the proportion of times the edge was oriented in the depicted direction (from → to). Figure S1: Centrality measures for anxiety and depression among bereaved mothers. Figure S2: Bridge centrality measures for anxiety and depression among bereaved mothers. Figure S3: Network stability (e.g., strength) for anxiety and depression symptoms by case‐dropping subset bootstrap. Figure S4: Bootstrap confidence intervals for edge weights. Each line represents the variability (95% CI) of an edge weight across 1000 bootstrap samples, with stronger edges showing narrower intervals and higher stability. Figure S5: Bootstrapped difference tests (α = 0.05) results between the node strength of depression and anxiety symptoms. (Note: The black boxes indicate a significant difference between the nodes, and the gray boxes indicate no significant difference between the nodes. White boxes show the values of node strength). [file DA-2026-9965609-s001.docx]

Supplementary materials for **“Symptom structure of depression and anxiety in mothers following child loss: A network and Bayesian graph analysis”**

Firoj Al-Mamun^1,2*^, Mohammed A. Mamun^1,2^, Moneerah Mohammad ALmerab^3^, Suzanne Holroyd^4^, David Gozal^5^, and Mohammad Muhit ^1^

1. Department of Public Health, University of South Asia, Dhaka 1348, Bangladesh [FAM: [firojphiju@gmail.com](mailto:firojphiju@gmail.com); MM: [mmuhit@hotmail.com](mailto:mmuhit@hotmail.com)]
2. CHINTA Research Bangladesh, Savar, Dhaka 1342, Bangladesh [MAM: [mamun@thechinta.org](mailto:mamun@thechinta.org)]
3. Department of Psychology, College of Education and Human Development, Princess Nourah bint Abdulrahman University, Riyadh, Saudi Arabia [MMA: [mmalmreab@pnu.edu.sa](mailto:mmalmreab@pnu.edu.sa)]
4. Department of Psychiatry and Behavioral Medicine, Joan C. Edwards School of Medicine, Marshall University, 1600 Medical Center Drive, Huntington, WV 25701, United States of America [SH: [holroyds@marshall.edu](mailto:holroyds@marshall.edu)]
5. Office of The Dean and Department of Pediatrics, Joan C. Edwards School of Medicine, Marshall University, 1600 Medical Center Dr, Huntington, WV 25701, United States of America [DG: [gozal@marshall.edu](mailto:gozal@marshall.edu)]

**Correspondence**

Firoj Al-Mamun, University of South Asia, Dhaka 1348 Bangladesh. Email: [firojphiju@gmail.com](mailto:firojphiju@gmail.com); [firoj@southasiauni.ac.bd](mailto:firoj@southasiauni.ac.bd)

Supplementary Table S1: Polychoric correlation matrix of anxiety and depressive symptoms among bereaved mothers (n = 2,276)

|  | A1 | A2 | A3 | A4 | A5 | A6 | A7 | D1 | D2 | D3 | D4 | D5 | D6 | D7 | D8 | D9 |
| --- | --- | --- | --- | --- | --- | --- | --- | --- | --- | --- | --- | --- | --- | --- | --- | --- |
| A1 | 1.0000 | 0.6869 | 0.6534 | 0.5663 | 0.4710 | 0.4085 | 0.4105 | 0.4753 | 0.5622 | 0.4052 | 0.4841 | 0.3937 | 0.4507 | 0.3677 | 0.3995 | 0.4089 |
| A2 | 0.6869 | 1.0000 | 0.6578 | 0.7379 | 0.6096 | 0.4472 | 0.4959 | 0.4730 | 0.5584 | 0.4224 | 0.4244 | 0.4324 | 0.4964 | 0.4080 | 0.4942 | 0.4759 |
| A3 | 0.6534 | 0.6578 | 1.0000 | 0.6939 | 0.5752 | 0.4175 | 0.4950 | 0.4489 | 0.5323 | 0.4048 | 0.4054 | 0.3888 | 0.4999 | 0.3780 | 0.4980 | 0.4771 |
| A4 | 0.5663 | 0.7379 | 0.6939 | 1.0000 | 0.7126 | 0.4935 | 0.5869 | 0.5068 | 0.5846 | 0.4317 | 0.4357 | 0.4952 | 0.5507 | 0.4774 | 0.5987 | 0.5856 |
| A5 | 0.4710 | 0.6096 | 0.5752 | 0.7126 | 1.0000 | 0.5015 | 0.6376 | 0.4908 | 0.5315 | 0.4210 | 0.4173 | 0.4295 | 0.5395 | 0.4618 | 0.5722 | 0.5863 |
| A6 | 0.4085 | 0.4472 | 0.4175 | 0.4935 | 0.5015 | 1.0000 | 0.4806 | 0.4667 | 0.4537 | 0.3901 | 0.3588 | 0.4030 | 0.4202 | 0.3933 | 0.4622 | 0.4561 |
| A7 | 0.4105 | 0.4959 | 0.4950 | 0.5869 | 0.6376 | 0.4806 | 1.0000 | 0.4677 | 0.5498 | 0.4166 | 0.3387 | 0.4567 | 0.5877 | 0.4608 | 0.5674 | 0.6256 |
| D1 | 0.4753 | 0.4730 | 0.4489 | 0.5068 | 0.4908 | 0.4667 | 0.4677 | 1.0000 | 0.6289 | 0.4508 | 0.5066 | 0.4323 | 0.5124 | 0.4548 | 0.4378 | 0.4846 |
| D2 | 0.5622 | 0.5584 | 0.5323 | 0.5846 | 0.5315 | 0.4537 | 0.5498 | 0.6289 | 1.0000 | 0.4735 | 0.5264 | 0.4643 | 0.5531 | 0.4796 | 0.5288 | 0.4957 |
| D3 | 0.4052 | 0.4224 | 0.4048 | 0.4317 | 0.4210 | 0.3901 | 0.4166 | 0.4508 | 0.4735 | 1.0000 | 0.5763 | 0.5784 | 0.4071 | 0.4306 | 0.4763 | 0.4282 |
| D4 | 0.4841 | 0.4244 | 0.4054 | 0.4357 | 0.4173 | 0.3588 | 0.3387 | 0.5066 | 0.5264 | 0.5763 | 1.0000 | 0.5727 | 0.4149 | 0.4017 | 0.3896 | 0.3469 |
| D5 | 0.3937 | 0.4324 | 0.3888 | 0.4952 | 0.4295 | 0.4030 | 0.4567 | 0.4323 | 0.4643 | 0.5784 | 0.5727 | 1.0000 | 0.4578 | 0.4676 | 0.5088 | 0.4653 |
| D6 | 0.4507 | 0.4964 | 0.4999 | 0.5507 | 0.5395 | 0.4202 | 0.5877 | 0.5124 | 0.5531 | 0.4071 | 0.4149 | 0.4578 | 1.0000 | 0.6052 | 0.6196 | 0.6289 |
| D7 | 0.3677 | 0.4080 | 0.3780 | 0.4774 | 0.4618 | 0.3933 | 0.4608 | 0.4548 | 0.4796 | 0.4306 | 0.4017 | 0.4676 | 0.6052 | 1.0000 | 0.6233 | 0.5596 |
| D8 | 0.3995 | 0.4942 | 0.4980 | 0.5987 | 0.5722 | 0.4622 | 0.5674 | 0.4378 | 0.5288 | 0.4763 | 0.3896 | 0.5088 | 0.6196 | 0.6233 | 1.0000 | 0.6937 |
| D9 | 0.4089 | 0.4759 | 0.4771 | 0.5856 | 0.5863 | 0.4561 | 0.6256 | 0.4846 | 0.4957 | 0.4282 | 0.3469 | 0.4653 | 0.6289 | 0.5596 | 0.6937 | 1.0000 |
| Note: Polychoric correlations were estimated to account for the ordinal (0-3) response format of PHQ-9 and GAD-7 items; Anxiety symptoms are labeled A1-A7, and depression symptoms D1-D9; All correlations are positive and statistically significant (*p* <0.001) | | | | | | | | | | | | | | | | |

Supplementary Table S2: Adjacency (edge-weight) matrix from EBICglasso network of anxiety and depression symptoms among bereaved mothers (n = 2,276)

|  | A1 | A2 | A3 | A4 | A5 | A6 | A7 | D1 | D2 | D3 | D4 | D5 | D6 | D7 | D8 | D9 |
| --- | --- | --- | --- | --- | --- | --- | --- | --- | --- | --- | --- | --- | --- | --- | --- | --- |
| A1 | 0.000 | 0.341 | 0.293 | -0.018 | -0.025 | 0.041 | -0.011 | 0.044 | 0.135 | 0.000 | 0.137 | 0.000 | 0.014 | 0.000 | -0.042 | 0.000 |
| A2 | 0.341 | 0.000 | 0.114 | 0.335 | 0.091 | 0.027 | 0.000 | 0.000 | 0.046 | 0.020 | 0.000 | 0.000 | 0.019 | 0.000 | 0.000 | 0.000 |
| A3 | 0.293 | 0.114 | 0.000 | 0.257 | 0.058 | 0.000 | 0.022 | 0.000 | 0.029 | 0.019 | 0.000 | -0.012 | 0.066 | -0.026 | 0.039 | 0.000 |
| A4 | -0.018 | 0.335 | 0.257 | 0.000 | 0.279 | 0.041 | 0.038 | 0.012 | 0.056 | 0.000 | 0.000 | 0.067 | 0.000 | 0.000 | 0.096 | 0.059 |
| A5 | -0.025 | 0.091 | 0.058 | 0.279 | 0.000 | 0.103 | 0.237 | 0.045 | 0.000 | 0.000 | 0.032 | -0.002 | 0.016 | 0.000 | 0.055 | 0.079 |
| A6 | 0.041 | 0.027 | 0.000 | 0.041 | 0.103 | 0.000 | 0.098 | 0.137 | 0.019 | 0.049 | 0.000 | 0.046 | 0.000 | 0.018 | 0.057 | 0.030 |
| A7 | -0.011 | 0.000 | 0.022 | 0.038 | 0.237 | 0.098 | 0.000 | 0.000 | 0.130 | 0.027 | -0.073 | 0.069 | 0.148 | 0.000 | 0.025 | 0.196 |
| D1 | 0.044 | 0.000 | 0.000 | 0.012 | 0.045 | 0.137 | 0.000 | 0.000 | 0.290 | 0.048 | 0.145 | 0.000 | 0.087 | 0.059 | -0.043 | 0.065 |
| D2 | 0.135 | 0.046 | 0.029 | 0.056 | 0.000 | 0.019 | 0.130 | 0.290 | 0.000 | 0.035 | 0.130 | 0.000 | 0.080 | 0.033 | 0.051 | 0.000 |
| D3 | 0.000 | 0.020 | 0.019 | 0.000 | 0.000 | 0.049 | 0.027 | 0.048 | 0.035 | 0.000 | 0.265 | 0.247 | 0.000 | 0.043 | 0.077 | 0.013 |
| D4 | 0.137 | 0.000 | 0.000 | 0.000 | 0.032 | 0.000 | -0.073 | 0.145 | 0.130 | 0.265 | 0.000 | 0.262 | 0.017 | 0.028 | -0.010 | -0.053 |
| D5 | 0.000 | 0.000 | -0.012 | 0.067 | -0.002 | 0.046 | 0.069 | 0.000 | 0.000 | 0.247 | 0.262 | 0.000 | 0.010 | 0.084 | 0.081 | 0.031 |
| D6 | 0.014 | 0.019 | 0.066 | 0.000 | 0.016 | 0.000 | 0.148 | 0.087 | 0.080 | 0.000 | 0.017 | 0.010 | 0.000 | 0.239 | 0.117 | 0.166 |
| D7 | 0.000 | 0.000 | -0.026 | 0.000 | 0.000 | 0.018 | 0.000 | 0.059 | 0.033 | 0.043 | 0.028 | 0.084 | 0.239 | 0.000 | 0.246 | 0.081 |
| D8 | -0.042 | 0.000 | 0.039 | 0.096 | 0.055 | 0.057 | 0.025 | -0.043 | 0.051 | 0.077 | -0.010 | 0.081 | 0.117 | 0.246 | 0.000 | 0.311 |
| D9 | 0.000 | 0.000 | 0.000 | 0.059 | 0.079 | 0.030 | 0.196 | 0.065 | 0.000 | 0.013 | -0.053 | 0.031 | 0.166 | 0.081 | 0.311 | 0.000 |

Supplementary Table S3. Centrality and Predictability of anxiety and depression symptoms among bereaved mothers (n = 2,276)

| Symptom | Strength | Closeness | Betweenness | Expected Influence | Predictability |
| --- | --- | --- | --- | --- | --- |
| A1 – Nervousness | 1.102 | 0.00570 | 34 | 0.909 | 0.670 |
| A2 – Uncontrollable worry | 0.992 | 0.00579 | 24 | 0.992 | 0.758 |
| A3 – Excessive worry | 0.937 | 0.00558 | 2 | 0.861 | 0.693 |
| A4 – Trouble relaxing | 1.258 | 0.00606 | 42 | 1.222 | 0.798 |
| A5 – Restlessness | 1.021 | 0.00581 | 26 | 0.967 | 0.773 |
| A6 – Irritability | 0.665 | 0.00446 | 0 | 0.665 | 0.667 |
| A7 – Feeling afraid | 1.075 | 0.00650 | 36 | 0.906 | 0.837 |
| D1 – Anhedonia | 0.976 | 0.00549 | 12 | 0.890 | 0.709 |
| D2 – Sadness | 1.034 | 0.00609 | 14 | 1.034 | 0.708 |
| D3 – Sleep disturbance | 0.842 | 0.00481 | 0 | 0.842 | 0.676 |
| D4 – Low energy | 1.153 | 0.00564 | 30 | 0.881 | 0.693 |
| D5 – Appetite change | 0.911 | 0.00522 | 8 | 0.882 | 0.706 |
| D6 – Guilt | 0.980 | 0.00507 | 10 | 0.980 | 0.832 |
| D7 – Poor concentration | 0.857 | 0.00477 | 6 | 0.805 | 0.835 |
| D8 – Psychomotor disturbance | 1.249 | 0.00542 | 14 | 1.058 | 0.875 |
| D9 – Suicidal ideation | 1.084 | 0.00529 | 8 | 0.979 | 0.924 |


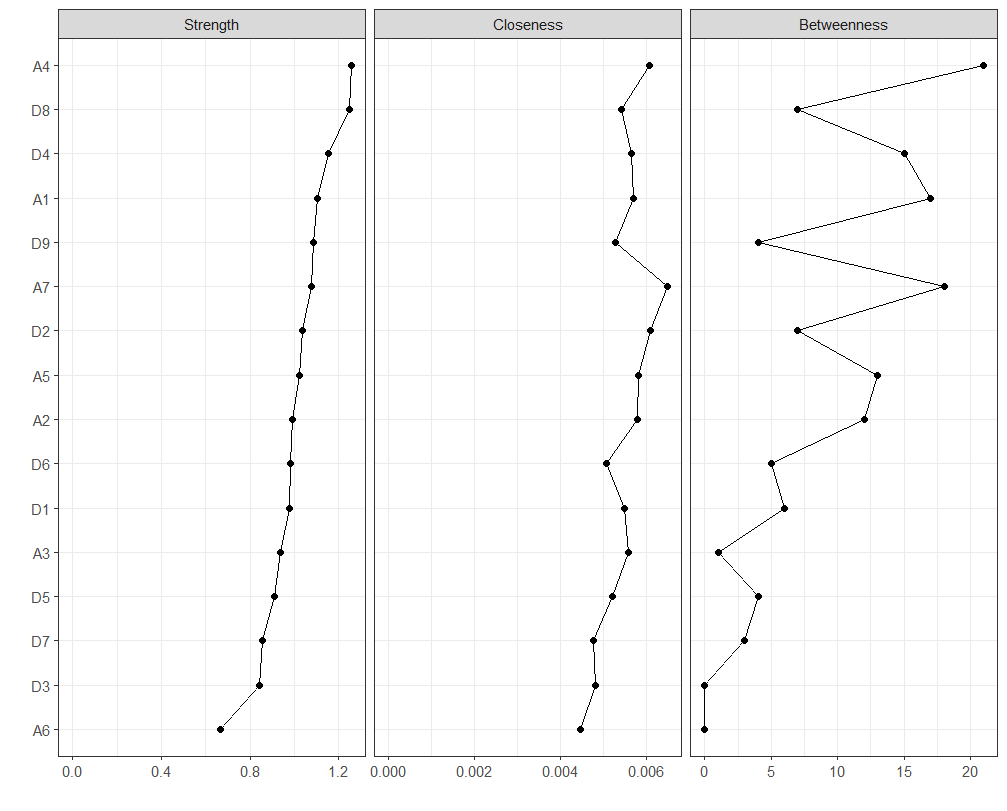


**Supplementary Figure S1:** Centrality measures for anxiety and depression among bereaved mothers


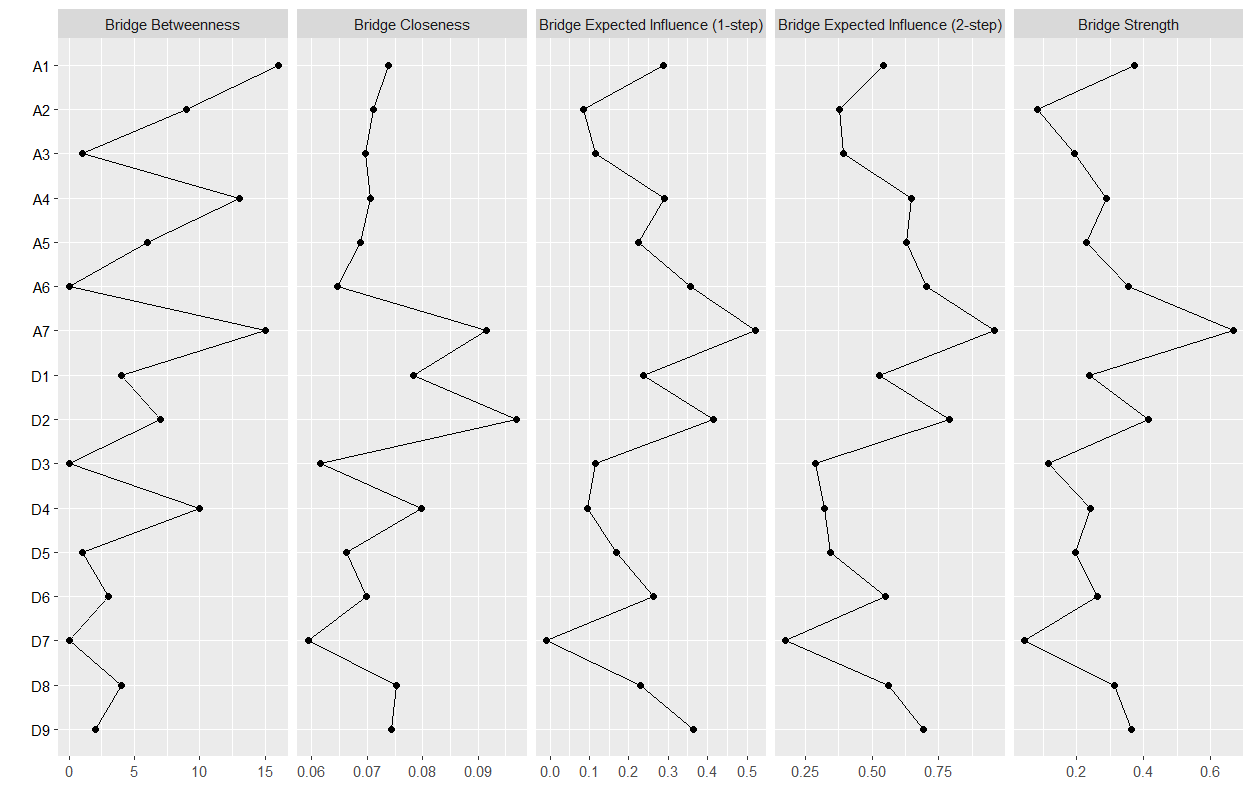


**Supplementary Figure S2:** Bridge centrality measures for anxiety and depression among bereaved mothers

Supplementary Table S4. Bridge centrality metrics for anxiety and depression symptoms among bereaved mothers (n = 2,276)

| Symptom | Bridge Strength | Bridge Betweenness | Bridge Closeness | Bridge EI (1-step) | Bridge EI (2-step) |
| --- | --- | --- | --- | --- | --- |
| A1 – Nervousness | 0.373 | 16 | 0.0739 | 0.288 | 0.543 |
| A2 – Uncontrollable worry | 0.084 | 9 | 0.0710 | 0.084 | 0.379 |
| A3 – Excessive worry | 0.192 | 1 | 0.0696 | 0.116 | 0.391 |
| A4 – Trouble relaxing | 0.290 | 13 | 0.0706 | 0.290 | 0.646 |
| A5 – Restlessness | 0.229 | 6 | 0.0687 | 0.224 | 0.631 |
| A6 – Irritability | 0.356 | 0 | 0.0646 | 0.356 | 0.706 |
| A7 – Feeling afraid | 0.669 | 15 | 0.0915 | 0.522 | 0.960 |
| D1 – Anhedonia | 0.238 | 4 | 0.0783 | 0.238 | 0.529 |
| D2 – Sadness | 0.416 | 7 | 0.0970 | 0.416 | 0.789 |
| D3 – Sleep disturbance | 0.115 | 0 | 0.0616 | 0.115 | 0.287 |
| D4 – Low energy | 0.243 | 10 | 0.0797 | 0.096 | 0.323 |
| D5 – Appetite change | 0.196 | 1 | 0.0663 | 0.167 | 0.345 |
| D6 – Guilt | 0.263 | 3 | 0.0698 | 0.263 | 0.550 |
| D7 – Poor concentration | 0.045 | 0 | 0.0593 | −0.008 | 0.176 |
| D8 – Psychomotor disturbance | 0.314 | 4 | 0.0752 | 0.229 | 0.563 |
| D9 – Suicidal ideation | 0.364 | 2 | 0.0744 | 0.364 | 0.693 |


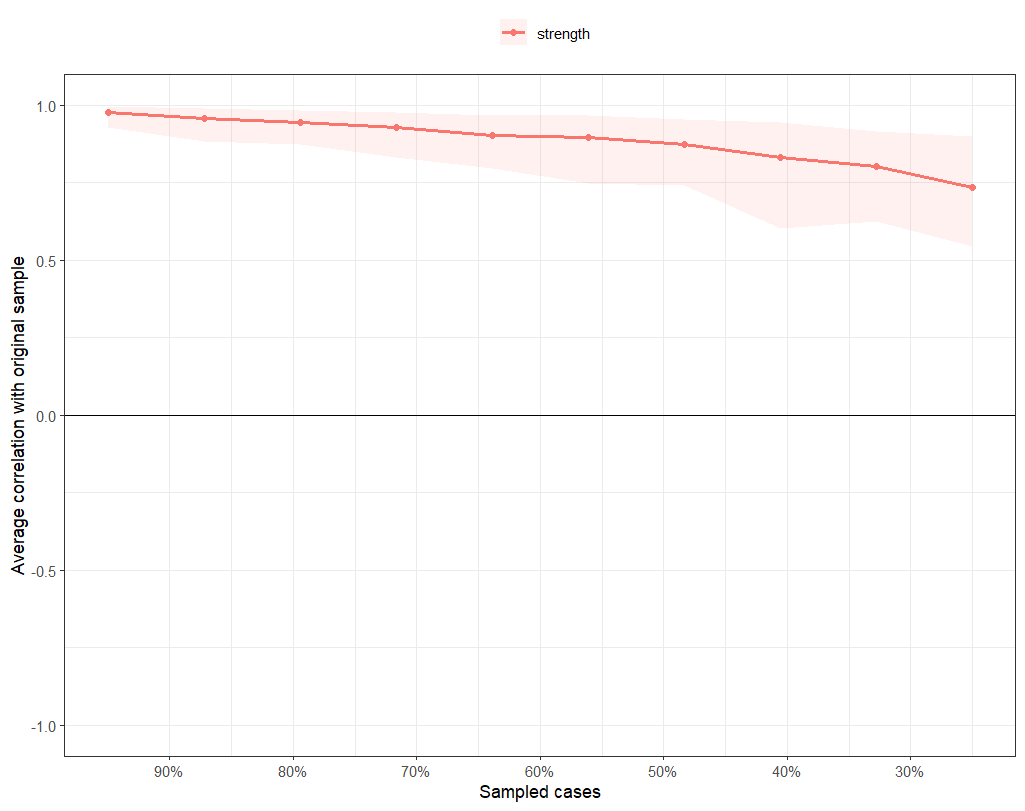


**Supplementary Figure S3:** Network stability (e.g., Strength) for anxiety and depression symptoms by case-dropping subset bootstrap


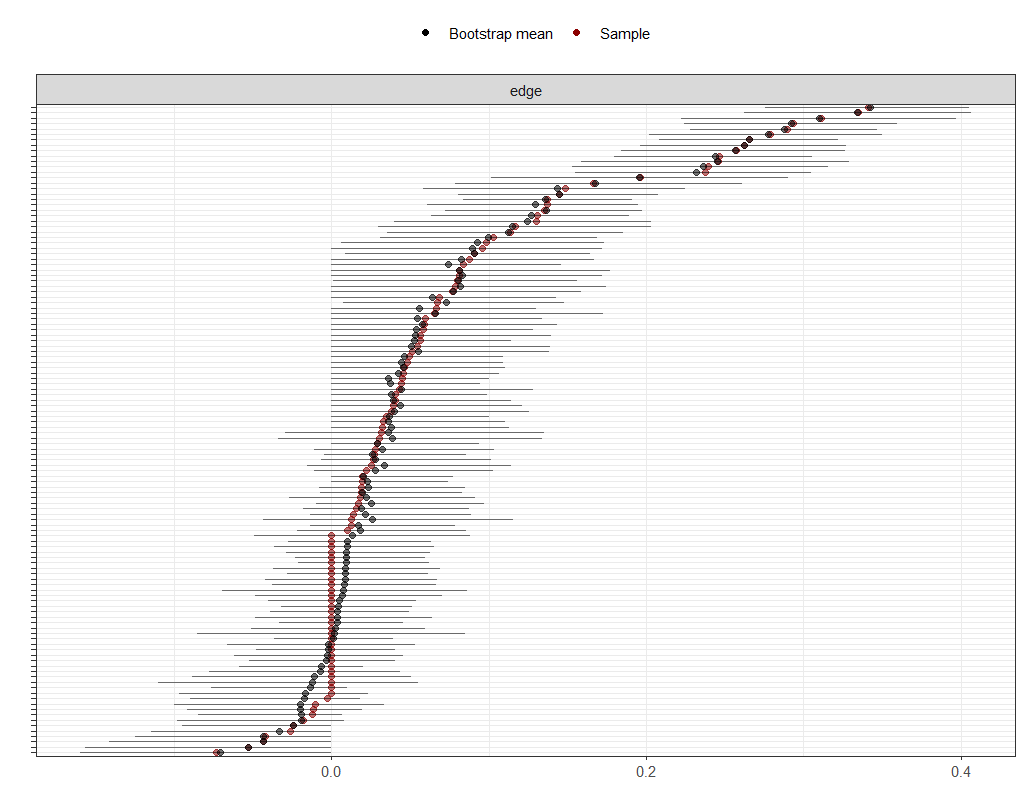


**Supplementary Figure S4:** Bootstrap confidence intervals for edge weights. Each line represents the variability (95% CI) of an edge weight across 1,000 bootstrap samples, with stronger edges showing narrower intervals and higher stability.


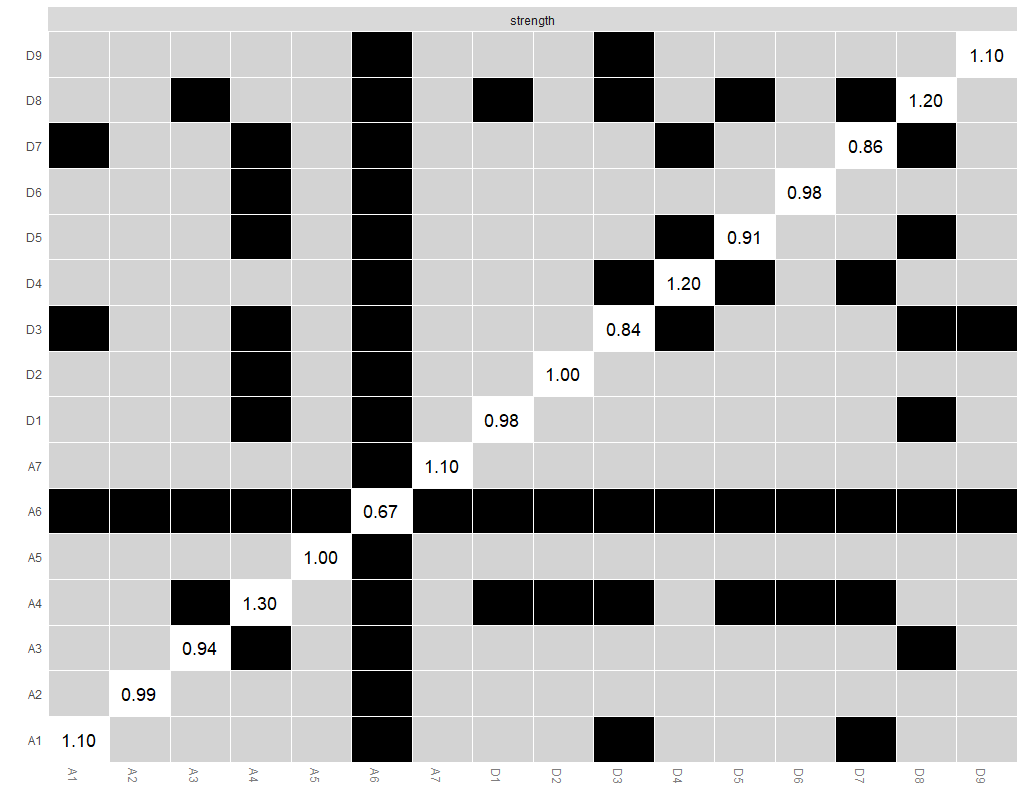


**Supplementary Figure S5:** Bootstrapped difference tests (α = 0.05) results between the node strength of depression and anxiety symptoms. [Note: The black boxes indicate a significant difference between the nodes, the gray boxes indicate no significant difference between the nodes. White boxes show the values of node strength].

**Supplementary Table S5:** Strongest directed edges in the Bayesian network (DAG) estimated from bootstrapped hill-climbing algorithm (bnlearn), ordered by edge frequency (strength > 0.85). For each edge, strength represents the proportion of bootstrap samples in which the edge appeared, and direction indicates the proportion of times the edge was oriented in the depicted direction (from → to).

| From | To | Strength | Direction (→ To) |
| --- | --- | --- | --- |
| A1 | A2 | 0.9999 | 0.5151 |
| A1 | A3 | 0.9831 | 0.8170 |
| A2 | A1 | 0.9999 | 0.4849 |
| A2 | A4 | 0.9998 | 0.4524 |
| A3 | A1 | 0.9831 | 0.1830 |
| A3 | A4 | 0.9637 | 0.1731 |
| A4 | A2 | 0.9998 | 0.5476 |
| A4 | A3 | 0.9637 | 0.8269 |
| A4 | A5 | 1.0000 | 0.5158 |
| A5 | A4 | 1.0000 | 0.4843 |
| A5 | A7 | 0.9587 | 0.5196 |
| A7 | A5 | 0.9587 | 0.4804 |
| D1 | D2 | 1.0000 | 0.3661 |
| D2 | D1 | 1.0000 | 0.6340 |
| D3 | D4 | 0.9954 | 0.4439 |
| D3 | D5 | 0.8760 | 0.5336 |
| D4 | D3 | 0.9954 | 0.5561 |
| D4 | D5 | 0.9503 | 0.6216 |
| D5 | D3 | 0.8760 | 0.4664 |
| D5 | D4 | 0.9503 | 0.3784 |
| D8 | D9 | 0.9362 | 0.5493 |
| D9 | D8 | 0.9362 | 0.4507 |
